# Kinetic analysis and structural studies of a high‐efficiency laccase from *Cerrena* sp. RSD1

**DOI:** 10.1002/2211-5463.12459

**Published:** 2018-07-03

**Authors:** Meng‐Hsuan Wu, Cheng‐Chung Lee, An‐Shan Hsiao, Su‐May Yu, Andrew H.‐J. Wang, Tuan‐Hua David Ho

**Affiliations:** ^1^ Institute of Plant and Microbial Biology Academia Sinica Taipei Taiwan; ^2^ Department of Life Sciences National Cheng Kung University Tainan Taiwan; ^3^ Institute of Biological Chemistry Academia Sinica Taipei Taiwan; ^4^ Institute of Molecular Biology Academia Sinica Taipei Taiwan; ^5^ Agricultural Biotechnology Center National Chung Hsing University Taichung Taiwan; ^6^ Department of Life Sciences National Chung Hsing University Taichung Taiwan

**Keywords:** basidiomycete laccase, diffusion‐limited enzyme, substrate‐binding loop

## Abstract

A high‐efficiency laccase, DLac, was isolated from *Cerrena* sp. RSD1. The kinetic studies indicate that DLac is a diffusion‐limited enzyme. The crystal structure of DLac was determined to atomic resolution, and its overall structure shares high homology to monomeric laccases, but displays unique substrate‐binding loops from those in other laccases. The substrate‐binding residues with small side chain and the short substrate‐binding loop IV broaden the substrate‐binding cavity and may facilitate large substrate diffusion. Unlike highly glycosylated fungal laccases, the less‐glycosylated DLac contains one highly conserved glycosylation site at N432 and an unique glycosylation site at N468. The *N*‐glycans stabilize the substrate‐binding loops and the protein structure, and the first *N*‐acetylglucosamine is crucial for the catalytic efficiency. Additionally, a fivefold increase in protein yield is achieved via the submerged culture method for industrial applications.

**Database:**

The atomic coordinates of the structure of DLac from *Cerrena* sp. RSD1 and structural factors have been deposited in the RCSB Protein Data Bank (PDB ID: 5Z1X).

**Abbreviations**
2.6‐DMP: 2,6‐dimethoxyphenol
ABTS: 2,2′‐azino‐bis(3‐ethylbenz‐thiazoline‐6‐sulfonic acid)
Endo H: endoglycosidase H
GlcNAc: acetylglucosamine
ITS: internal transcribed spacer
PDB: Protein Data Bank
PNGase F: peptide‐*N*‐glycosidase F
RBBR: Remazol Brilliant Blue R
SBL: substrate‐binding loop

Laccases are important biocatalysts for a number of applications, including delignification, bioremediation, biosensor, and analytical applications 1, and have been studied continually and extensively. H_2_O_2_‐independent laccase is more eco‐friendly than other lignin‐modifying enzymes because it only requires O_2_ and produces water as the by‐product of catalysis 2, 3. Laccase can be found in bacteria, insects, fungi, and plants, and fungal laccases show higher redox potentials than other laccases 4, 5, 6, 7. At least sixty fungal strains have been reported to have laccase activities, including ascomycetes, deuteromycetes, and basidiomycetes 8. In white‐rot basidiomycetes, laccases are used to oxidize phenolic compounds, leading to the degradation of lignin 9. 2,2′‐Azino‐bis(3‐ethylbenz‐thiazoline‐6‐sulfonic acid) (ABTS) is a common substrate of laccases as well as a mediator that can extend its substrate range from phenolic compounds to nonphenolic substrates 10. However, the difficulty of the large‐scale production of fungal laccases hinders their industrial usage because of the long generation times of fungi and multiple enzyme isoforms, as well as complicated purification steps. Even though the heterologous expression is a promising technique, the expression of fungal laccases in *Escherichia coli* has not been reported 11. The protein yield and laccase activity obtained from the expression of fungal laccase in *Pichia pastoris* are considerably low compared to the native forms due to differences in the glycosylation patterns 12, 13.

Fungal laccases typically have 3 to 10 glycosylation sites, and 10 to 50% of their molecular weight is attributed to glycosylation 1, 14, 15. The deglycosylation of laccase in *Pycnoporus sanguineus* affects its enzyme kinetics, and the loop–glycan interaction is crucial for laccase function in *Lentinus* sp. 12, 16. However, structural data are limited due to low resolution and the high flexibility of glycan molecules. Previous studies have indicated that the loss of laccase activity in *Rigidoporus lignosus* and *Pleurotus ostreatus* is associated with the protein conformation rather than with the coordination of metal site geometry at the active site 17. A portion of the structure of *Rigidoporus lignosus* laccase is sensitive to high temperatures, which leads to the loss of enzyme activity 18. Many studies have focused on the improvement of the enzyme activity of laccase via protein engineering approaches, such as random mutation 19, site‐directed mutation 20, and directed evolution 21. Most of the mutations on the surface and in loop regions have been suggested to increase enzyme activity 21, 22. The active site and the copper coordination geometry are highly conserved among laccases. Therefore, the residues on the protein surfaces and in loop regions must play a crucial role in improving the enzyme activity. Unfortunately, these prior studies lacked either kinetic data or structural evidence to justify how these residues affect the laccase activity.

The main focus of this study was to discover a highly active laccase and to understand what structural features governing the enzyme kinetics. A unique laccase called DLac was isolated from *Cerrena* sp. RSD1. It displays high catalytic efficiency in the diffusion‐limited range compared to all previously studied enzymes. A productive submerge culture system was established for DLac production, purification, and crystallization. The crystal structure was determined to atomic resolution to investigate the correlations between the structural properties and the catalytic efficiency. The observed glycosylation also implicates its important role in the stabilization of substrate‐binding loops (SBLs) and domain 3 (D3).

## Results and Discussion

### Identification of DLac from *Cerrena* sp. RSD1

**Figure 1:**
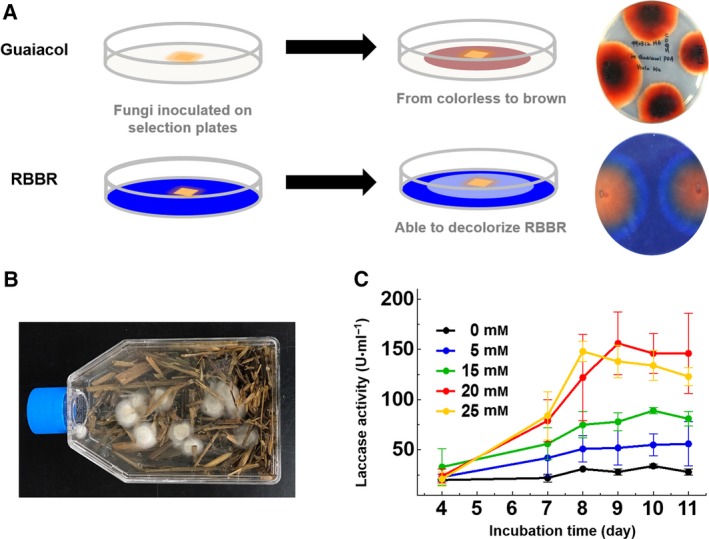
Screening of fungi from rice straw compost and optimization of the culture conditions for enzyme production. (A) Substrate‐containing plates for screening high‐activity laccase‐secreting fungi. Fungi were inoculated onto the guaiacol plate (upper panel) and RBBR plate (lower panel) to distinguish the laccase activity directly by visible color change. (B) The submerged culture method was developed for enzyme production. (C) The effect of the inducer 2,5‐dimethylaniline was evaluated in flask‐scale enzyme production. Different concentrations of 2,5‐dimethylaniline were used, from 0 to 25 mm. Error bars represent standard deviation.

**Figure 2:**
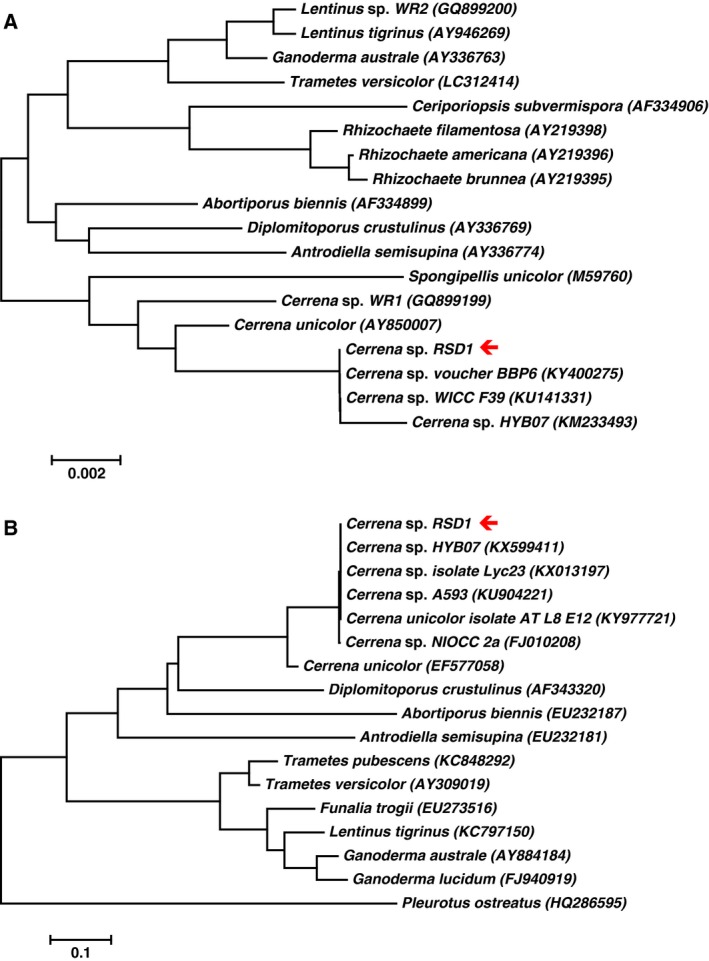
Phylogenetic analysis based on 18S rDNA sequence and ITS sequence alignment. The molecular phylogenies of *Cerrena* sp. RSD1 (arrow) and basidiomycete species were compared with 18S sequence (A) and ITS sequence (B) data from GenBank using BLASTn search. The numbers in parentheses are accession numbers of the sequences. The sequences were aligned using Clustal W, and phylogenetic evolutionary analyses were conducted using MEGA6.06 by the neighbor joining method.

**Figure 3:**
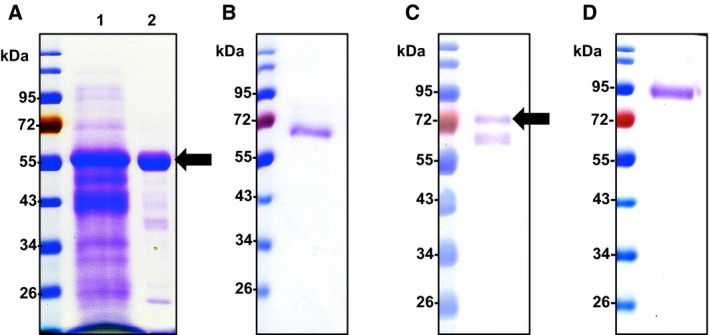
Enzyme purity verified by SDS/PAGE. (A) DLac. Lane 1 is the crude filtrate from the fungal culture, and lane 2 is the purified DLac (arrow). (B) TvLac. (C) AbLac (arrow). (D) MtLac.

**Figure 4:**
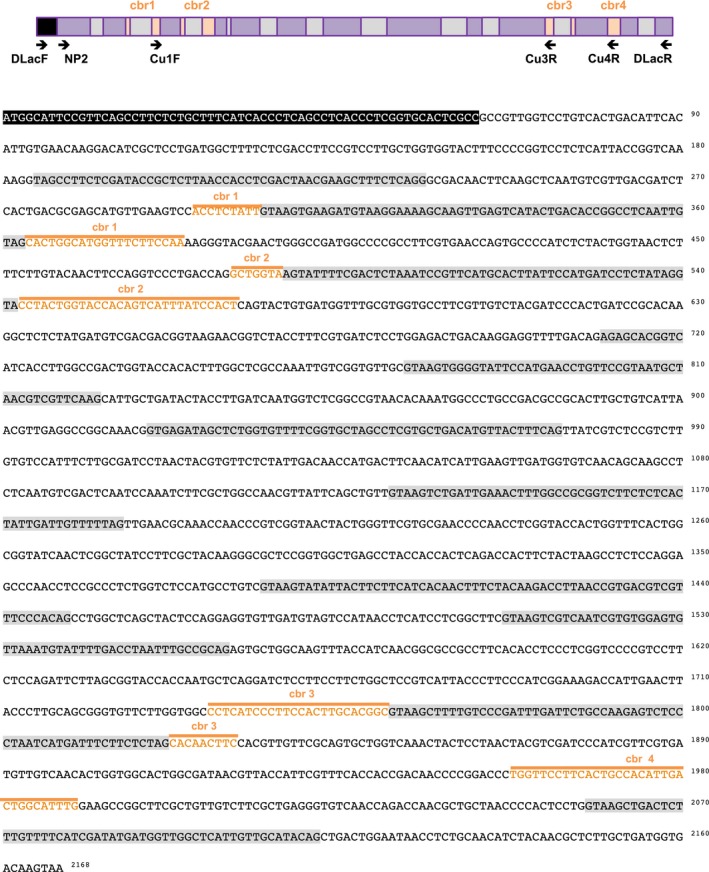
Gene map of *DLac*. The *DLac* gene contains 11 introns (gray) and 12 exons. The signal peptide is colored in a black background, and four copper‐binding sites (cbr1‐4) are colored in orange.

As high‐efficiency fungal laccases are required for industrial applications, Taiwanese indigenous fungi were isolated from rice straw composts from Shan‐hua in Taiwan. The dominant microbes in the degrading compost, which revealed lignocellulosic enzyme activity for rice straw degradation, are well adapted to growth under low pH and high temperature conditions. To screen for fungi producing high‐efficiency laccases, we have developed a high‐throughput screening method (Fig. 1A) based on the color change on fungi‐cultured plates containing guaiacol or Remazol Brilliant Blue R (RBBR) 23, 24. A fungal strain exhibiting the strongest laccase activity was isolated, and the laccase secreted from a submerged culture of the *Cerrena* sp. RSD1 was designated DLac. Phylogenetic analysis using 18S rDNA sequence and internal transcribed spacer (ITS) sequences indicated that the *Cerrena* sp. RSD1 belongs to the genus *Cerrena* (Fig. 2). The crude filtrate of *Cerrena* sp. RSD1 and purified DLac were analyzed by SDS/PAGE (Fig. 3A). The purified DLac protein band observed on the gel was subsequently isolated for *N*‐terminal amino acid sequencing. The *N*‐terminal sequence ‘AVGPVTDI’ and highly conserved copper‐binding regions were used to design the degenerate primers for DLac gene sequencing. The full‐length *DLac* contains 2168 bp with a 1551‐bp coding sequence encoding 516 amino acids, including a signal peptide of 21 amino acids (Fig. 4). The mature protein has 495 amino acids with a calculated molecular mass of 52.9 kDa. NCBI BLAST analysis indicated that the gene with highest homology to DLac is the laccase 7 precursor of *Cerrena* sp. HYB07 (GenBank accession no. KF317949). The cDNA and amino acid sequences shared 98.6 and 100% identity, respectively 25.

### Production, purification, and characterization of DLac

**Figure 5:**
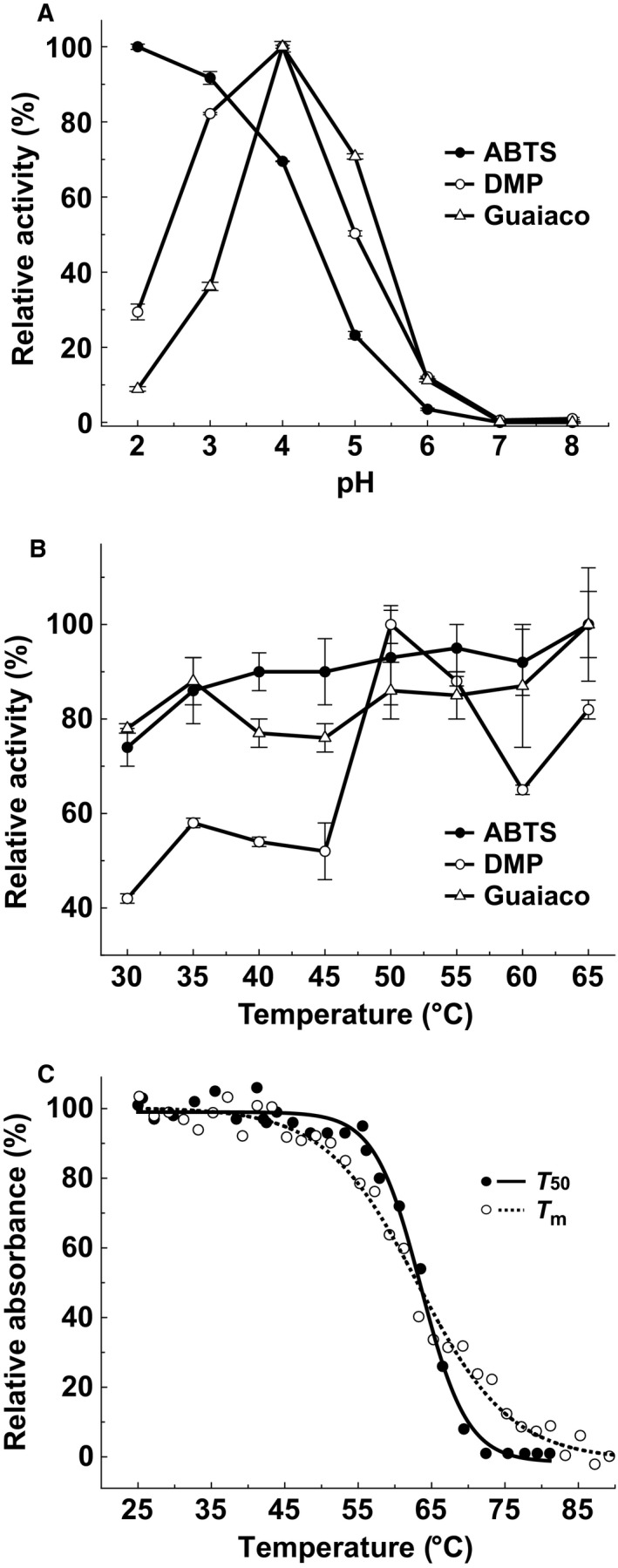
Optimal pH, optimal temperature, and thermostability of DLac. The pH (A) and temperature (B) effects on DLac were determined in a standard enzymatic activity assay using different substrates, including ABTS (closed circle), DMP (open circle), and guaiacol (open triangle). The activities of DLac at 65 °C for ABTS and guaiacol and at 50 °C for DMP were defined as 100%. Error bars represent standard deviation. (C) Thermostability investigation of DLac. The *T*_50_ curve (closed circle with solid line) was plotted using the residual enzyme activities after 10 minutes of heating from 25 to 81 °C. The *T*_m_ curve (open circle with dashed line) was detected by CD.

Here, we used a submerged culture to produce DLac and its production was fivefold enhanced in an 8‐day culture by adding 2,5‐dimethylaniline as an inducer (Fig. 1B–C). The utilization of 2,5‐dimethylaniline as an inducer has also been observed in the production of other basidiomycete laccases 26, 27. The crude filtrate was collected, and the DLac protein was further purified using anion‐exchange and size‐exclusion chromatography. We then characterized the chemical properties of DLac using ABTS, 2,6‐dimethoxyphenol (2,6‐DMP) and guaiacol as substrates. The optimal pH value for ABTS is pH 3.0, and pH 4.0 is the optimal pH reaction value for DMP and guaiacol (Fig. 5A). Both ABTS and guaiacol have optimal temperatures of 65 °C, and that for DMP is 50 °C (Fig. 5B). The kinetic parameters of DLac were also determined using ABTS, DMP, and guaiacol as substrates. DLac has the highest turnover rate, substrate affinity, and catalytic efficiency for ABTS, with a *k*_cat_ of 52 515 s^−1^, a *K*_M_ of 36 μm, and a *k*_cat_/*K*_M_ of 1.5 × 10^9^ s^−1^·m ^−1^ (Table 1). The catalytic efficiency was higher than 10^8^ s^−1^·m ^−1^, suggesting that DLac is a diffusion‐limited enzyme 28. We further compared the kinetic parameters of DLac with those of other fungal laccases, which have high oxidation efficiency of ABTS. The *k*_cat_ of DLac is higher than those of other fungal laccases with the exceptions of POXA3a and POXA3a from *Pleurotus ostreatus*, while its *K*_M_ value is average.

**Table 1:** Comparison of kinetic parameters of fungal laccases

Laccase	PDB	*k*_cat_(s^−1^)	*K*_M_(μm)	*k*_cat_/*K*_M_(s^−1^·m ^−1^)	Condition	References
*Pleurotus ostreatus* POXA3b^a^	–	158 333	74	2.1 × 10^9^	pH 3.6, 25 °C	36
*Pleurotus ostreatus* POXA3a^a^	–	73 333	70	1.0 × 10^9^	pH 3.6, 25 °C	36
DLac^a^	5Z1X	52 515	36	1.5 × 10^9^	pH 3.0, 65 °C	This study
		35 568	24	1.5 × 10^9^	pH 3.0, 30 °C	This study
DLac^b^		8	57	1.4 × 10^5^	pH 4.0, 50 °C	This study
DLac^c^		1	641	1.6 × 10^3^	pH 4.0, 65 °C	This study
TvLac^a^	1GYC	26 803	38	6.0 × 10^8^	pH 3.0, 65 °C	This study
		22 391	28	6.0 × 10^8^	pH 3.0, 30 °C	This study
MtLac^a^	–	17 590	12	1.5 × 10^9^	pH 3.0, 65 °C	This study
		13 883	4	3.1 × 10^9^	pH 3.0, 30 °C	This study
*Lentinus* sp.^a^	3X1B	3382	65	5.2 × 10^7^	pH 2.5, 70 °C	12
*Tricholoma mongolicum* a	–	1480	2	6.4 × 10^8^	pH 4.5, 30 °C	53
AbLac^a^	–	7885	134	5.9 × 10^7^	pH 3.0, 65 °C	This study
		5560	212	2.6 × 10^7^	pH 3.0, 30 °C	This study
*Pleurotus pulmonarius* a	–	1520	210	7.2 × 10^6^	pH 4.5, 40 °C	54
*Rigidoporus lignosus* a	1V10	1360	200	6.8 × 10^6^	pH 3.0, 25 °C	55
*Trametes pubescens* a	–	690	14	4.8 × 10^7^	pH 3.0, 25 °C	56
*Meripilus giganteus* a	–	546	17	3.7 × 10^7^	pH 3.0, 30 °C	57
*Trametes hirsuta* a	3FPX	196	41	4.8 × 10^6^	pH 5.0, 25 °C	35

a Kinetic parameters were measured using ABTS as substrate.

b Kinetic parameters were measured using DMP as substrate.

c Kinetic parameters were measured using guaiacol as substrate.

–, not determined.

As ABTS is the mediator for laccase‐mediator system applications, we have chosen this substrate to determine the thermostability of DLac. The *T*_50_ and *T*_m_ values of DLac are 63.3 °C and 62.9 °C that is close to its optimal temperature 65 °C, respectively (Fig. 5C). The heat‐induced conformational change increases the enzyme activity of DLac, similar to a combination of SDS and heat‐induced bacterial laccase from *Azospirillum lipoferum* after catalytic activation 29. Interestingly, two thermostable basidiomycete laccases, laccase Lac‐4.8 from *Physisporinus rivulosus* and laccase I from basidiomycete PM1, showed thermal activation after preheating 23, 30, 31, suggesting that thermally induced transitions in laccases are relevant to enzyme activation. In contrast, previous studies have indicated that the optimal temperature for several enzymes is much lower than their *T*_m_ 32, 33, 34.

To study the kinetic comparison between DLac and other laccases, we further purified three commercial laccases, *Trametes versicolor* laccase (TvLac), *Agaricus bisporus* laccase (AbLac), and recombinant *Myceliophthora thermophila* laccase (MtLac) (Fig. 3B–D), and compared their kinetics parameters for ABTS oxidation (Table 1). Different from AbLac, the catalytic efficiencies of DLac, TvLac, and MtLac were higher than 10^8^ s^−1^·m ^−1^, suggesting that these laccases are also considered to be diffusion‐limited enzymes. Our results further indicate that, when the temperature increases from 30 to 65 °C, both the *k*_cat_ and *K*_M_ of DLac, TvLac, and MtLac increase (Table 1). This is consistent with previous findings that the substrate diffusion rate is the factor determining the catalytic efficiency of diffusion‐limited enzymes 28. Notably, using ABTS as the substrate, fungal laccases show catalytic efficiencies in a range from μm ^−1^·s^−1^ to nm ^−1^·s^−1^ 35, 36. The produced DLac with a high enzyme activity for ABTS oxidation displays high potential for industrial applications.

### Overall structure

**Figure 6:**
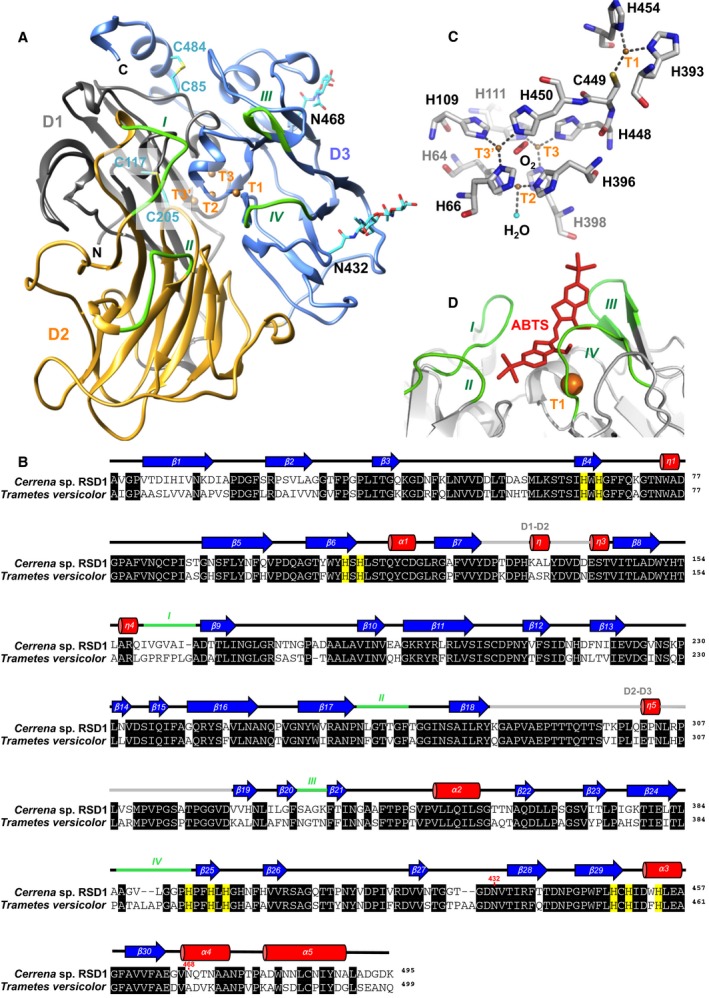
Crystal structure of DLac. (A) Cartoon representation of the DLac crystal structure. D1, D2, and D3 are indicated in gray, yellow, and blue; the SBLs (*I*,*II*,*III,* and *IV*) in D2 and D3 are shown in green. The T1, T2, T3, and T3′ copper atoms are represented by orange spheres. The carbohydrates and disulfide bonds are represented by stick models. (B) Protein sequence from DLac aligned with TvLac. Residues in α‐helices, β‐strands, and loops are shown as red cylinders, blue arrows, and black lines, respectively. The loops involved in substrate binding are marked with green lines, and the linkage between domains is marked with gray lines and labeled the D1‐D2 or D2‐D3. The glycosylation sites are marked with red numbers on the amino acid sequence. (C) Three types of copper‐binding sites in DLac. The copper atoms T1, T2, T3, and T3′ are coordinated by the surrounding histidine, cysteine, oxygen molecule, and water molecule. Protein residues and the oxygen molecule are shown as stick models, and the oxygen atoms are shown in red, nitrogen in blue, sulfur in yellow, and carbon in gray. The four copper ions and the water molecules are represented by orange and cyan spheres, respectively. (D) The substrate‐binding cavity on the protein surface of DLac is docked with the substrate, ABTS. The ABTS molecule was taken from the substrate‐binding site of laccase soaked with ABTS from *Bacillus subtilis* (PDB: 1OF0). The SBLs, ABTS, and T1Cu are represented in green, red, and orange, respectively.

To investigate the relationship between the structure and the activity of DLac, the crystal structure of DLac was determined to a resolution of 1.38 Å (Fig. 6A). The DLac crystals belong to space group *P*2_1_2_1_2_1_, with two molecules in an asymmetric unit. The current structure was refined to an *R*_work_ of 12.6% and an *R*_free_ of 15.0% (Table 2). The overall structure of DLac is similar to the crystal structure of TvLac and consists of thirty β‐strands, five α‐helices, and five 3_10_‐helices (Fig. 6B), which fold into three cupredoxin‐like domains: domain 1 (D1, 1–127), domain 2 (D2, 142–281), and domain 3 (D3, 324–495). Each domain folds into a Greek key β‐barrel topology. The D1 and D2 domains are linked by the D1‐D2 loop (128–141), and the D2 and D3 domains are connected by the D2‐D3 loop (282–323). Moreover, the structure is further stabilized by two disulfide bridges. The D1 and D2 domains are connected by a disulfide bond between C117 and C205, and D1 and D3 are also linked through a disulfide bridge between C85 and C484 (Fig. 6A). Three potential *N*‐glycosylation sites, N432, N468, and N474, were predicted by the NetNGlyc 1.0 Server 25. However, only two *N*‐glycosylation sites, N432 and N468 in D3, were observed in the crystal structure of DLac (Fig. 6A). Both chains A and B observed in the crystal structure display identical glycosylation patterns.

**Table 2:** Data collection and refinement statistics^a^

Protein	DLac
Data collection	
Wavelength (Å)	1.0
Space group	*P* 2_1_2_1_2_1_
Cell dimensions (Å)	*a* = 85.63, *b* = 88.80, *c* = 147.82
Resolution (Å)	30.0–1.38 (1.43–1.38)
Unique reflections	228 137
*R*_merge_ (%)	4.2 (27.2)
*I*/σ(*I*)	44.2 (4.8)
Completeness	98.6 (95.0)
Redundancy	4.3 (2.7)
Refinement	
Resolution (Å)	25.0–1.38
No. of reflections *R*_work_/*R*_free_	216 276/11 402
*R*_work_/*R*_free_	12.6/15.0
No. of atoms/Avg B factor (Å^2^)	
Protein	7488/15.3
Glycan	84/25.4
Copper ion	8/14.9
Other	1654/33.3
RMSD	
Bond lengths (Å)	0.004
Bond angles (°)	1.16
Ramachandran statistics (%)^b^	
Ramachandran favored	97.97
Ramachandran outliers	0.41

a Values corresponding to the highest resolution shell are shown in parentheses.

b The stereochemistry of the model was validated with MolProbity 52.

### Active site and functional loops

The active site of DLac consists of four copper atoms. A mononuclear T1 copper (T1Cu) is localized between D2 and D3, and a T2/T3 trinuclear cluster, including one T2 copper and two T3 coppers (T3 and T3′), is located between D1 and D3 (Fig. 6A). The T1Cu is coordinated by H393, C449, and H454, and the T2/T3 trinuclear cluster is bound by eight highly conserved histidine residues in laccases (Fig. 6C). The T2 copper is coordinated by H64 and H396 and an axial water molecule, while each T3 copper is coordinated to three histidines (H111, H398, and H448 for the T3 copper and H66, H109, and H450 for the T3′ copper) and one mutual oxygen molecule. The substrate‐binding cavity of DLac was determined by superimposition with the laccase soaked with ABTS from *Bacillus subtilis* (Fig. 6D). Therefore, the SBLs of DLac were identified as loop I: 159–165, loop II: 264–270, loop III: 332–337, and loop IV: 386–393.

### Comparative analysis of the sequence and structure among laccases

**Figure 7:**
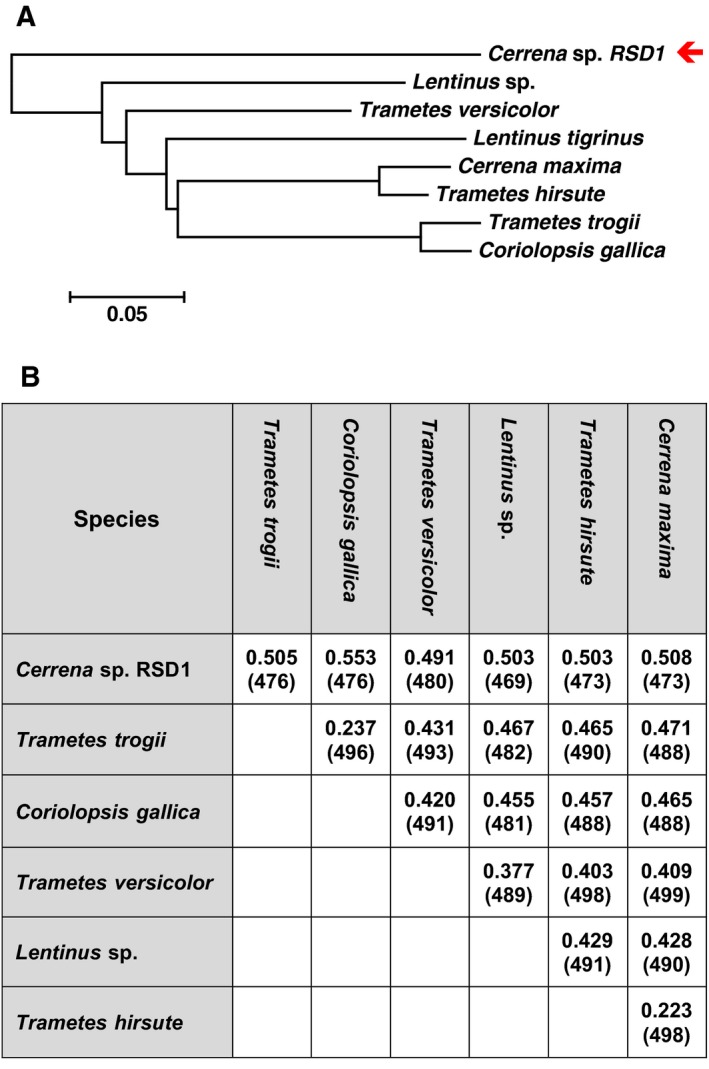
Phylogenetic sequence analysis and structure alignment of laccases. (A) Phylogenetic analysis of basidiomycete laccases based on amino acid sequence alignment. The sequences were aligned using Clustal W, and phylogenetic evolutionary analyses were conducted using MEGA6.06 by the neighbor joining method. (B) The RMSD matrix and the number of aligned Cα residues from the superimposed basidiomycete laccase structure alignment. The protein sequences and crystal structure of DLac from *Cerrena* sp. RSD1 (PDB: 5Z1X) were compared with those of basidiomycete laccases, including *Trametes trogii* (PDB: 2HRG), *Coriolopsis gallica* (PDB: 4A2E), *Trametes versicolor* (PDB: 1GYC), *Lentinus* sp. (PDB: 3X1B), *Trametes hirsute* (PDB: 3FPX), and *Cerrena maxima* (PDB: 3DIV).

**Figure 8:**
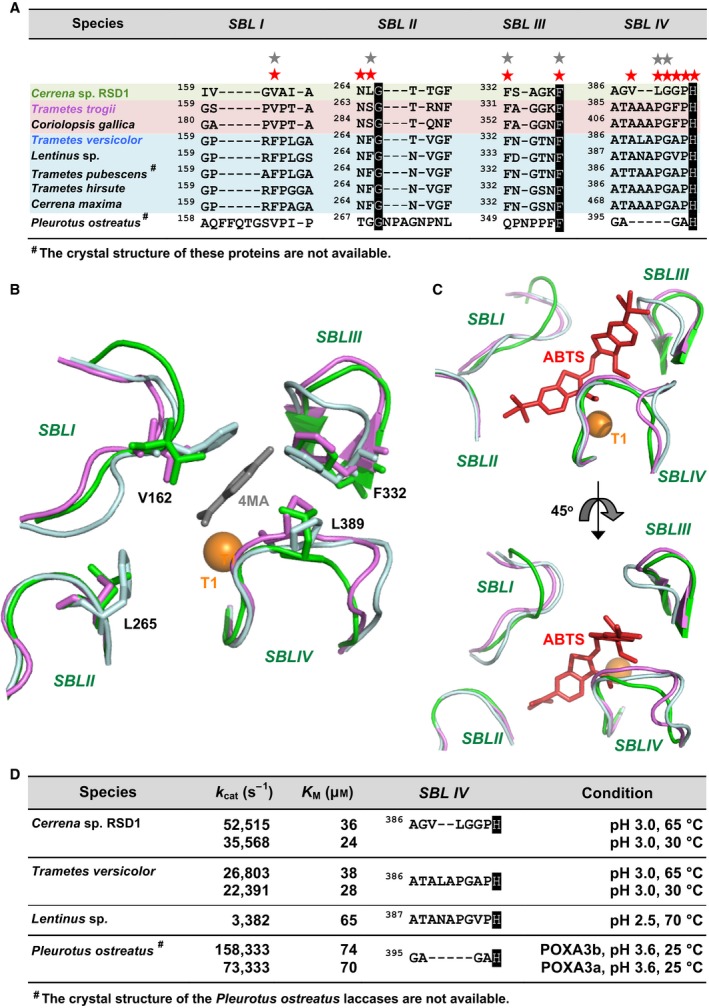
Structure‐based sequence alignment of SBLs in laccases from basidiomycetes. (A) Sequences of SBLs were compared between laccases from *Cerrena* sp. RSD1, *Trametes trogii*,* Coriolopsis gallica*,* Trametes versicolor*,* Lentinus* sp.*, Trametes pubescens*,* Trametes hirsute, Cerrena maxima,* and *Pleurotus ostreatus*. Residues conserved in all laccases are shown in black. Nonpolar amino acids providing hydrophobic interactions with 4MA are labeled with gray stars. The amino acids involving interactions with ABTS are labeled with red stars. Loops of the laccases from *Cerrena* sp. RSD1, *Trametes trogii,* and *Trametes versicolor* are shown in green, pink, and cyan, and their corresponding side chains are compared in (B). The superimposition of the SBLs of laccases is docked with 4MA (gray) and ABTS (red) in Fig. 8B and 8C, respectively. The 4MA was taken from the active site of the laccase complex with 4MA in *Trametes trogii* (PDB: 2HRG). A series of hydrophobic residues (V162, L265, F332, and L389) involved in substrate binding are indicated. The ABTS was taken from the active site of the laccase complex with ABTS in *Bacillus subtilis* (PDB: 1OF0). The T1Cu is shown as an orange sphere. (D) Kinetic parameters and sequences of SBL IV were compared between laccases from *Cerrena* sp. RSD1, *Trametes versicolor*,* Lentinus* sp., and *Pleurotus ostreatus*.

**Figure 9:**
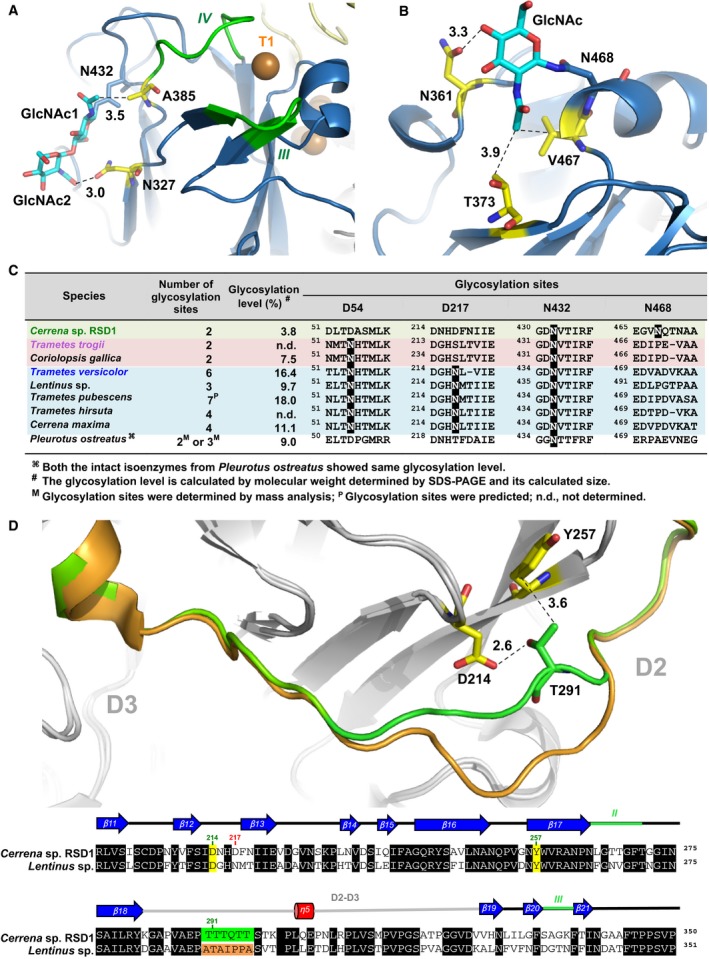
The glycosylation structure of DLac and the effect of deglycosylation on DLac. (A) Two GlcNAc residues (cyan) on N432 and the contact residues (yellow) are represented in a stick model. The dashed lines indicate interactions between *N*‐glycans and the surrounding amino acid residues. Substrate‐binding loops III and IV (green) and the T1 copper (orange) are shown. (B) The mono‐GlcNAc residue (cyan) on N468 and its interactions with amino acid residues (yellow) are shown in a stick model. The dashed lines indicate interactions between the glycan and the surrounding residues. (C) The *N*‐glycosylation sites of basidiomycete laccases were aligned by structure‐based superimposition. The glycosylation sites of laccases from nine species of basidiomycetes, including *Cerrena* sp. RSD1, *Trametes trogii*,* Coriolopsis gallica*,* Trametes versicolor*,* Lentinus* sp.*, Trametes pubescens*,* Trametes hirsute, Cerrena maxima,* and *Pleurotus ostreatus*, were analyzed. *N*‐linked glycosylation sites are shown in black. (D) The D2‐D3 loops of DLac (green) and *Lentinus* sp. laccase (orange) were superimposed. The residues D214 (yellow), Y257 (yellow), and T291 (green) in DLac are represented in a stick model. The protein sequence from DLac aligned with *Lentinus* sp. laccase. Residues in η‐helices, β‐strands, and loops are shown as red cylinders, blue arrows, and black lines, respectively. The loops involved in substrate binding are marked with green lines, and the linkage between domains is marked with gray lines and labeled the D2‐D3. The residues at 217 and residues at 214, 257, and 291 are marked with red and green numbers on the amino acid sequence, respectively. The TTTQTT region in DLac and its corresponding region in *Lentinus* sp. laccase are colored in green and orange background, respectively.

The protein sequence and crystal structure of DLac were compared with the known structures of basidiomycete laccases, including TvLac and laccases from *Trametes trogii* 37, *Coriolopsis gallica* 38, *Lentinus* sp. 12, *Trametes hirsute* 35, and *Cerrena maxima* 39 (Figs 7, 8, 9). The phylogenetic tree using the primary amino acid sequence of DLac locates it far from other basidiomycete laccases (Fig. 7A). To determine the precise differences between DLac and basidiomycete laccases, a structural superimposition was performed to calculate the root‐mean‐square deviation (RMSD) between the structures. Besides DLac, the structures of basidiomycete laccases are highly similar with a RMSD of 0.223 ~ 0.471 Å for 481 ~ 499 Cα atoms. However, DLac is distinguished from other laccases with an RMSD of 0.491 ~ 0.553 Å for 469 ~ 480 Cα atoms (Fig. 7B). To further identify the unique feature in the crystal structure of DLac, it was compared in detail to that of TvLac, which shows the highest sequence identity and the lowest RMSD value relative to DLac. Sequence alignment between DLac and TvLac indicates that different fragments appear in the *N*‐terminal, *C*‐terminal, and dispersed loops (Fig. 6B). The observation of conservation of the active site geometry but with differences in the loop combinations is consistent with previous studies 7, 14. Among the loops, we highlight the SBLs as targets, which directly affect the substrate diffusion into active site. All SBLs are different in either length or amino acid composition (Fig. 6B). Both DLac and TvLac show similar *K*_M_ values, while a difference occurs in *k*_cat_ (Table 1). We thus suggest that the difference in SBLs between DLac and TvLac may play an important role in determining the kinetic properties.

### Substrate‐binding loops of DLac

We further compared the SBL regions of DLac to other basidiomycete laccases (Fig. 8A). According to length and similarity of sequence, we classified SLB into three patterns. The heterodimeric laccases, POXA3a and POXA3b, from *Pleurotus ostreatus* which displays highest activity 36 were also included by sequence alignment result of their large subunit that is identical between isoforms 40. The SBL IV in DLac and *Pleurotus ostreatus* laccases are shorter than those in others, with deletion of two and five amino acids, respectively. Additionally, the SBL IV of DLac is rich in glycine in comparison with other laccases, providing more flexibility at the active site, similar to a previous study on HIV‐1 integrase, which showed higher enzyme activity after glycine‐to‐alanine substitutions on the active site loop 41. To identify the functional residues involved in substrate binding, the crystal structure of DLac was superimposed with that of laccases from *Trametes trogii* and *Bacillus subtilis* complexed with their substrates, methylbenzoic acid (4MA, Fig. 8B) and ABTS (Fig. 8C), respectively. The nonpolar residues V162, L265, F332, F337, L389, and G390 in the SBLs interact with the 4MA by hydrophobic contact. L389 on loop IV of DLac supplies a larger side chain than the corresponding proline residue in other laccases. Additional five residues, N264, V388, G391, P392, and H393 of DLac, are involved in ABTS‐binding. Among these residues, the side chains of V162, L265, and G391 of DLac are smaller than the corresponding residues from other laccases. Previous studies on TvLac suggested that nonpolar amino acids with smaller side chains give more space in the binding cavity for the entry of larger substrates 42, 43. The contact between large substrate, ABTS, and SBL IV is much closer than that of small substrate 4MA. DLac and *Pleurotus ostreatus* laccases containing substrate‐binding residue 162V, and shorter loop IV show higher *k*_cat_ than that of other laccases (Fig. 8D). In contrast to *Pleurotus ostreatus* laccases, the V388, L389, and G390 on loop IV of DLac may contribute to increase ABTS‐binding affinity. Moreover, the kinetic study of DLac indicated the substrate affinity of small substrates (DMP and guaiacol) is lower than that of large substrate (ABTS) (Table 1). This property is consistent with prior studies of *Pleurotus ostreatus* laccases 36. In view of the fact that the turnover rate of diffusion‐limited enzymes depends on the diffusion rate of substrates, we suggest that the smaller side chains of V162, L389, and G341 and the shorter loop IV of DLac generates a larger cavity for substrate entry, which may facilitate the diffusion and catalysis of substrates.

### Functional analysis of *N*‐glycans in the crystal structure of DLac

Although the SBLs were similar, the glycosylation plays another important role in determining the kinetic property, for example, isoforms from *Pleurotus ostreatus* that display identical SBLs but show difference between glycosylation leads to twofold difference in *k*_cat_ 36, 40. Here, the glycosylation site of DLac is another distinctive feature from other laccases. Two asparagine residues (N432 and N468) were glycosylated with a disaccharide and a monosaccharide, respectively (Fig. 6A). The first *N*‐acetylglucosamine (*N*‐GlcNAc) on N432 has hydrophobic interaction with the side chain of A385, which is contiguous to the SBL IV, mainly stabilizing loop IV (Fig. 9A). The second GlcNAc on N432 interacts with the side chain of N327 by hydrogen bonding, which is extended by SBL III, indirectly stabilizing loop III. Therefore, the glycans attached to N432 result in stabilizing the substrate‐binding pocket. The function of the two‐first GlcNAcs on N432 in DLac is consistent with the study of *Lentinus* sp. laccase 12. The mono‐GlcNAc on N468 interacts with N361, T373, and V467 via hydrogen bonds and hydrophobic interactions (Fig. 9B). All these residues that interact with the mono‐GlcNAc on N468 are localized in D3, resulting in stabilization of the protein structure.

We further compared *N*‐glycosylation sites at position 54, 217, 432, and 468 between DLac and basidiomycete laccases (Fig. 9C). N432 is highly conserved in all basidiomycete laccases 12, while N468 is found only in DLac. Two *N*‐glycosylation sites, N54 and N217, commonly observed in basidiomycete laccases, were replaced by aspartic acids at D54 and D217 in DLac. D54 in DLac, which indicates that glycosylation at N54 is not important for enzyme activity, was also reported as nonessential for enzyme activity using site‐directed mutagenesis 12. Furthermore, the previous study also indicated the glycosylation at N217 in *Lentinus* sp. laccase plays an important role in stabilizing D2‐D3 loops 12. However, no glycosylation site at D217 was found in DLac. We thus superimposed the D2‐D3 loops between DLac and *Lentinus* sp. laccase (Fig. 9D). The result indicated the one amino acid shorter and threonine‐rich region, TTTQTT, in DLac was corresponding to ATAPPSA in *Lentinus* sp. laccase. The residue of T291 in DLac forms hydrogen bond with D214 and shares hydrophobic contact with Y257. Both D214 and Y257 are conserved between DLac and *Lentinus* sp. laccase. Additionally, the glycosylation level of DLac, which is relatively lower than other laccases (Fig. 9C), is estimated from the difference between the 55.0 kDa obtained from SDS/PAGE analysis and its calculated size of 52.9 kDa. All the *N*‐glycans detecting from the crystal structure of DLac functionally stabilize loop interactions in the substrate‐binding site and in D3, which differs from other basidiomycete laccases.

### Effect of glycosylation on the enzyme kinetics of DLac

**Figure 10:**
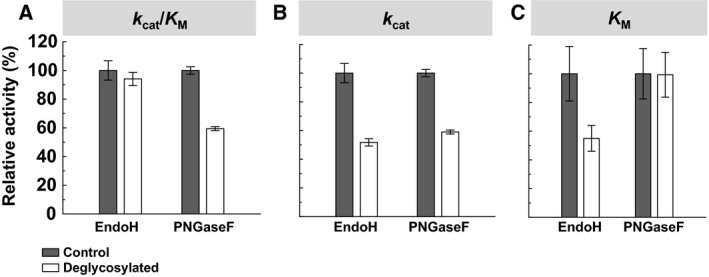
Kinetic studies of DLac after treatment with Endo H and PNGase F. The enzyme activity of DLac was measured in control conditions (gray) or after deglycosylation treatment (white). The kinetic parameters, including *k*_cat_/*K*_M_ (A), *k*_cat_ (B), and *K*_M_ (C), were compared independently. Error bars represent standard deviation.

To understand the effect of glycosylation on the kinetic efficiency, we deglycosylated DLac using treatment with endoglycosidase H (Endo H) and peptide‐*N*‐glycosidase F (PNGase F). The Endo H treatment, which leaves only one remaining GlcNAc on the glycosylation sites, had no significant effect on *k*_cat_/*K*_M_. In contrast, the catalytic efficiency decreased by 41% when all the glycan was cleaved by PNGase F (Fig. 10A). This result is consistent with the study of deglycosylation and site‐directed mutagenesis in *Lentinus* sp. laccase 12. Both Endo H and PNGase F deglycosylation led to similar decreases in the *k*_cat_ of DLac (Fig. 10B). The substrate affinity (45% decrease in *K*_M_) was increased by Endo H treatment, while it did not change with PNGase F treatment (Fig. 10C). We conclude that the presence of the first GlcNAc residue in DLac is essential for its high catalytic efficiency. Clarification of the functionality of glycans in DLac is helpful in choosing the proper heterologous expression system.

In conclusion, the high‐efficiency laccase, DLac, has been isolated from *Cerrena* sp. RSD1, and the protein yield was fivefold improved using a submerged culture in the presence of the inducer, 2,5‐dimethylaniline. This diffusion limited DLac consists of the small hydrophobic residues in SBL I, II, and IV and a short SBL IV, which provides a broad cavity for substrate‐binding, hence enhancing the diffusion rate of large substrates. Structural evidence also indicates its *N*‐glycans stabilize the SBLs III and IV and the D3. The maintenance of the first GlcNAc is essential for enzyme efficiency. This study presents the large substrate‐binding cavity, and the low glycosylation level of DLac contributes to its high efficiency to oxidize bulky substrate, ABTS, and these structural features can be used for protein engineering.

## Materials and methods

### Enzyme activity and kinetic studies

The standard laccase activity was determined by spectrophotometric measurements using the Synergy™ Mx Monochromator‐Based Multi‐Mode Microplate Reader (BioTek). The standard reaction mixture contains 198 μL of 0.5 mm ABTS (ε_420_ = 3.60 × 10^4^ m ^−1^·cm^−1^) in pH 3.0 McIlvaine's buffer and 2 μL of the enzyme solution. The enzyme assay was performed at 30 or 65 °C. One unit of enzyme activity was defined as 1 μmole of substrate transformed to product per minute 44. The kinetic study of DLac was performed using 1.25 ~ 800 μm ABTS at pH 3.0, 3.13 ~ 2000 μm DMP (ε_477_ = 1.48 × 10^4^ m ^−1^·cm^−1^) at pH 4.0, and 18.78 ~ 3000 μm guaiacol (ε_465_ = 1.2 × 10^4^ m ^−1^·cm^−1^) at pH 4.0. The initial rates were acquired from the linear portion of the experimental curve, and the kinetic parameters were determined using the Michaelis–Menten model in SigmaPlot 10.0.

### Submerged cultivation and laccase production

The *Cerrena* sp. RSD1 was cultured on 3.9% potato dextrose agar (PDA, Becton, Dickinson and Company) plates. To screen for the fungal strains exhibiting the highest laccase activity, we used PDA plates containing 0.04% guaiacol (Sigma#G5502) or RBBR (Sigma#R8001). For laccase production, twelve 4‐day‐old mycelial plugs (8 mm in diameter) were inoculated into 6.0 g of autoclaved rice straw submerged in 60 mL of minimal medium (10.0 g·L^−1^ glucose and 4.4 g·L^−1^ ammonium tartrate, pH 5.5) and 0.4 mm CuSO_4_. The fungal cultures were incubated in the bottles agitated at 100 r.p.m. and 30 °C for 16 days. On the fourth day, 20 mm 2,5‐dimethylaniline (Sigma#102253) was added as the inducer. The enzyme production was monitored by measuring the laccase activity in the supernatant.

### Cloning of the DLac sequence

Genomic DNA was extracted using the Wizard® Genomic DNA purification kit (Promega); total RNA was isolated with the TRIzol® Reagent (Ambion®). Total cDNA was labeled with an anchor sequence by RT‐PCR using SuperScript® III Reverse Transcriptase (Novagen) and the RT‐polyT primer. To clone the laccase gene from the *Cerrena* sp. RSD1, the cDNA of DLac was specifically amplified using the degenerated primer NP2. Other degenerate primers Cu1F, Cu3R, and Cu4R, which were designed from conserved copper‐binding regions 1, 3, and 4 in fungal laccases 45, were used for DLac sequencing. To obtain the signal peptide sequence, 5′‐RACE was performed with SMART II™ A oligonucleotide, the GSP1 primer and 5′ PCR Primer II A. The ORF and the full gene of DLac were amplified using primers DLacF and DLacR from the total cDNA or genomic DNA. All the PCRs in this study were amplified using TAKARA Ex Taq® DNA polymerase (TaKaRa Bio, Inc.) and performed in a T3000 Thermocycler (Biometra, Goettingen, Germany). The primers used for the PCR amplification are listed in Table S1.

### Purification of laccases and deglycosylation analysis

Rice straw was submerged in 20 mm Tris/HCl buffer (pH 8.0) at 4 °C overnight to wash out the enzyme absorbed on the rice straw. The supernatant was filtrated through INSIDE CéRAM minifilter MSKTB060010070‐0.14 μ (TAMI) and concentrated using a Xampler Ultrafiltration Cartridge (UFP‐10‐E‐4MA, GE Healthcare) with the QuixStand System. After concentration, DLac was purified with a HiTrap Q column and a HiPrep 16/60 Sephacryl S‐100 high‐resolution column. The purity of the protein was verified by SDS/PAGE. Three commercial laccases, including TvLac (#51639 and #38429), AbLac (#40452), and MtLac (#SAE0050), were purchased from Sigma‐Aldrich. All these commercial laccases were dialyzed in 20 mm Tris/HCl (pH 8.0) and purified by a HiTrap Q column and Superdex 75 10/300 GL column. All the laccases were stored in 20 mm Tris/HCl with 150 mm NaCl (pH 8.0). For deglycosylation assays, purified DLac was treated with either Endo H (New England BioLabs) or Remove‐iT™ PNGase F (New England BioLabs) for 24 h at 37 °C according to the manufacturer's instructions. The residual activity was measured by standard enzymatic assay.

### Effects of pH and temperature on the enzyme activity

The pH and temperature effects on the enzyme activity were evaluated by preparing the substrate suspensions in McIlvaine's buffer in the pH range of 2.2‐8.0 at 30 °C and in the temperature in the range of 30–65 °C at pH 3.0 for ABTS and at pH 4.0 for 2,6‐DMP and guaiacol. The *T*_50_ value was assessed in a gradient thermocycler (Biometra T Gradient Thermocycler), corresponding to the temperature at which the enzyme retains 50% of its activity after a 10‐min incubation. Subsequently, 176 μL aliquots were used in triplicate assays at temperatures between 25 and 82 °C. After a 10‐min incubation, samples were immediately chilled in ice water for 10 min. Thereafter, 2 μL of each sample (the protein concentration was 5 μg·mL^−1^) was used to measure the enzyme activity.

### Near‐UV circular dichroism (CD) spectropolarimetry

CD experiments were performed on a Jasco‐815 spectropolarimeter equipped with a six‐position Peltier temperature control system (PTC‐4245). The sample was prepared with 40 μm DLac in 4 mm Tris/HCl and 30 mm NaCl buffer (pH 8.0). The CD spectra were measured between 260 and 320 nm in a 1.0 mm light path length quartz cuvette with a step size of 1.0 nm. The signals at 280 nm were recorded during heating from 25 to 95 °C at 1 °C intervals and at the rate of 1 °C per minute.

### Crystallization and data collection

Crystals of the DLac were grown by mixing 1 μL of protein (15 mg·mL^−1^) with 1 μL of reservoir solution using the sitting‐drop vapor diffusion method at 20 °C. The DLac crystals were obtained in a reservoir solution of 1.4 m ammonium sulfate, pH 7.6. All the crystals were flash‐frozen with 20–25% glycerol as a cryoprotectant, and the diffraction patterns were recorded at cryogenic temperatures. The diffraction data were collected at a wavelength of 1.000 Å using the synchrotron BL15A beamline of NSRRC in Taiwan, with a MX300HE CCD detector. The diffraction data were processed and scaled using the program HKL2000 46.

### Structure determination and refinement

The DLac crystal structure was determined by molecular replacement using the program MOLREP of the CCP4 program suite 47, and the crystal structure of TvLac 48 (PDB ID: 1GYC) was used as a search model. Throughout the refinement, 5% of randomly selected data were set aside for cross‐validation with the *R*_free_ values. Manual modifications of the models were performed using the program Coot 49. Difference Fourier (*F*_o_‐*F*_c_) maps were calculated to locate the solvent molecules. All the crystal structures were refined using Refmac5 50, including the individual isotropic B‐factor refinement. The data collection and final model statistics are shown in Table 2. The molecular figures were produced using UCSF Chimera 51. The atomic coordinates and structure factors of the DLac crystal structure have been deposited in the PDB ID: 5Z1X.

## Author contributions

MHW conducted the design of work, cultivation, purification, biochemical characterization, kinetic study, and writing of the manuscript. CCL performed the crystallographic measurements, structure solution, and revision of the manuscript. ASH conducted the revision of the manuscript. SMY, AHJW, and THH revised the manuscript and supervised this manuscript.

## Supporting information


**Table S1**. Primers used for DLac cloning.
