# Supplementary material for: Kinetic analysis and structural studies of a high‐efficiency laccase from Cerrena sp. RSD1
Source: FEBS Open Bio. 2018 Jul 3;8(8):1230–46. doi: 10.1002/2211-5463.12459 (PMC6070645; doi:10.1002/2211-5463.12459)
Supplement: Supplementary file 1 — Table S1. Primers used for DLac cloning. [file FEB4-8-1230-s001.docx]

**Table S1 Primers used for DLac cloning.**

| **Primer** | **Sequence (5’ to 3’)** | **Purpose** |
| --- | --- | --- |
| RT-polyT | GGTTCTTGCCACAGTCACGAC(TTT)_6_ | RT-PCR |
| NP2 | GCNGTNGGNCCNGTNACNGAYAT | Amplification of cDNA of DLac |
| Cu1F | CAYTGGCAYGGNTTYTTYCA | Internal primer used for DLac sequencing |
| Cu3R | TGICCRTGIARRTGIANIGGRTG | Internal primer used for DLac sequencing |
| Cu4R | TGRAARTCDATRTGRCARTG | Internal primer used for DLac sequencing |
| SMART II^TM^ A oligonucleotide | AAGCAGTGGTATCAACGCAGAGTACGCGGG | To synthesize cDNA for 5’-RACE |
| GSP-1 | GTGGTACCAGTCGGCCAAGG | 5’-RACE |
| 5' PCR Primer II A | AAGCAGTGGTATCAACGCAGAGT | 5’-RACE |
| RT-anchor | GGTTCTTGCCACAGTCACGAC | 3’-RACE |
| DLacF | ATGGCATTCCGTTCAGCCTTCTC | Specific amplification of DLac gene from 5’ end |
| DLacR | TTACTTGTCACCATCAGCAAGAGCG | Specific amplification of DLac gene from 3’ end |
